# U.S. Immigration Policy Environment Contributions to Maternal and Child Health in the Latino Population

**DOI:** 10.3390/ijerph23030275

**Published:** 2026-02-24

**Authors:** Cynthia N. Lebron, Anna-Michelle McSorley, Vanessa Morales, Hannah T. Peterson, Veronica Morales

**Affiliations:** 1 School of Nursing and Health Studies, University of Miami, Coral Gables, FL 33146, USA; 2 Latino Caucus for Public Health in Affiliation with the American Public Health Association, Washington, DC 20001, USA; 3 Department of Allied Health Sciences, University of Connecticut, Storrs, CT 06269, USA; 4 Department of Public Health Sciences, University of Miami, Miami, FL 33146, USA; 5 Jacob D. Fuchsberg Law Center, Touro University, Central Islip, NY 11722, USA

**Keywords:** immigrant health, maternal and child health, public charge

## Abstract

**Highlights:**

**Public health relevance—How does this work relate to a public health issue?**

- U.S. immigration and public benefits policies directly shape access to health care, nutrition, and economic supports for Latino families, influencing maternal, infant, and early childhood health outcomes.
- Policy-driven barriers and “chilling effects” reduce participation in essential services during a critical developmental period with lifelong consequences for health and well-being.

**Public health significance—Why is this work of significance to public health?**

- Latino families represent a large and growing share of the U.S. population, meaning immigration-related policy changes have broad and long-term implications for national maternal and child health indicators.
- By synthesizing evidence across immigration, health, and social policy, this work highlights how structural and legal determinants, rather than individual behaviors, drive disparities in maternal and child health outcomes.

**Public health implications—What are the key implications or messages for practitioners, policy makers and/or researchers in public health?**

- Policies that expand eligibility and protect access to core safety-net programs for immigrant families can improve maternal and infant outcomes, reduce health disparities, and strengthen population health.
- Public health practitioners and researchers must consider immigration policy as a central determinant of health and advocate for policies that reduce fear, stabilize access to care, and center family well-being.

**Abstract:**

Latino families in the United States experience persistent maternal and child health (MCH) inequities driven by a fragmented immigration and public benefits policy environment rather than inherent health differences. Although most Latino children are U.S.-born citizens, many live in mixed-status families in which immigration status determines eligibility for health care, nutrition assistance, and other essential services. This narrative policy review examines U.S. immigration and public benefit policies from 1965 to 2025 to assess how eligibility rules, enforcement practices, and policy instability shape access to maternal and child health services among Latino populations. Drawing on public health, legal, and social science literature, the review documents substantial variation in access to Medicaid, CHIP, nutrition programs, and emergency care by immigration status and state policy. Findings indicate that restrictive eligibility criteria, expansions and contractions of the public charge rule, and immigration enforcement practices have produced chilling effects that deter eligible families from accessing care, reduce prenatal and postpartum service utilization, and contribute to adverse birth outcomes and intergenerational health inequities. The review concludes that immigration policy functions as a structural determinant of MCH and identifies two key policy priorities: 1. maintaining the 2022 Final Public Charge Rule that excludes public safety-net programs, and 2. waiving the five-year Medicaid waiting period for all pregnant immigrants regardless of documentation status to ensure equitable access to essential maternal and child health care.

## 1. Introduction

The United States maintains a fragmented policy environment for Latino families seeking maternal and child health (MCH) services. Notably, 18 million Latino children live in the United States, representing a substantial and rapidly growing share of the U.S. child population [1]. While Latino families are not inherently less healthy than other U.S. populations, they experience systemic legal, financial, linguistic, and social barriers that restrict access to the care they need [2,3]. For example, although 94% of Latino children are U.S.-born, nearly half have at least one parent born outside the United States. Families may include undocumented individuals, people with pending asylum or visa applications, lawful permanent residents subject to multi-year waiting periods for federal benefits, humanitarian entrants, or U.S. citizen children [4]. Understanding the maternal and child health experiences of Latino families requires recognizing the wide range of immigration statuses that exist in the United States, each carrying different rights, restrictions, and levels of access to health care and social services. For example, compared with U.S. citizens, immigrants (particularly noncitizens and non-permanent residents) had substantially lower insurance coverage and markedly worse access to preconception, prenatal, and postpartum healthcare, including fewer healthcare visits and a lower likelihood of having a usual source of care [5].

In this review, we seek to describe key elements of the ever-changing immigration policy landscape as they relate to complications surrounding immigration status and access to health care and public health services for women and children who identify as part of the Latino population in the U.S. We seek to elucidate the pathways by which the U.S. immigration policy environment, from 1965 to 2025, contributes to differing outcomes in maternal and child health for members of the Latino community. Our review begins with an overview of the policy mechanisms that shape access to MCH health care and public health services among Latinos in the U.S. We conclude with policy recommendations.

### 1.1. Public Benefit Eligibility

Persistent policy shifts under the current administration continue to complicate eligibility decisions for government-funded public services. This includes Medicaid, emergency Medicaid and the Child Health Insurance Program (CHIP)—a network of programs that help cover the cost of medical expenses for individuals with limited resources, including low-income families, women and children; the Supplemental Nutrition Assistance Program and Special Supplemental Nutrition Program for Women, Infants and Children (WIC)—programs that provide healthy food, nutrition education and breastfeeding support to families; and Temporary Assistance for Needy Families (TANF) and Supplemental Security Income (SSI)—sources of economic stability for low-income families or for individuals with disabilities [6,7]. In July 2025, we observed rescission of Medicaid and CHIP access for legally present immigrants, refugees, and asylees [8]. These benefits, previously in place to support families in our communities with documented status in the U.S., are no longer available. These continuing policy shifts, in combination with precarious immigration statuses, create an environment of confusion and instability as to which public benefits these non-citizens may access.

As presented in Table 1, there is substantial variation in access to benefits by immigration status [9,10]. Currently, United States citizens and lawful permanent residents are eligible for key public health benefits, whereas their non-citizen or visa holder counterparts are more limited. Notably, the variation among this already limited group is further differentiated depending on their immigrant category. Those with a clear pathway to a green card (i.e., family-sponsored visa holders, refugees, asylees, victims of certain abuse) are eligible for a greater number of benefits. Whereas individuals with no path to a green card (i.e., DACA recipients, TPS holders, withholding of removal recipients, and non-immigrant temporary visa holders) are granted lawful permission to remain in the United States, they remain limited in their eligibility for benefits, usually only permitted to access WIC and emergency Medicaid. Although some public insurance options exist for pregnant immigrants who are otherwise ineligible for Medicaid, these pathways are limited and uneven across states [11]. Emergency Medicaid is available nationwide but covers only labor and delivery, excluding prenatal and most postpartum care [12]. Indeed, eligibility varies not only by status but also by state policies and administrative discretion.

As of December 2025, 31 states have expanded pregnancy-related Medicaid coverage to certain immigrant groups, including waiving the five-year waiting period for pregnant permanent residents and extending coverage during pregnancy to low-income immigrants regardless of status; however, these policies are not universal, often exclude postpartum care beyond sixty days, do not address preconception coverage, and may be underutilized due to persistent fears of immigration-related consequences associated with public program enrollment [5,13]. Such coverage fragmentation is compounded by an increasingly hostile political climate surrounding immigration and a worsening national maternal health crisis [14]. Harmful stereotypes, xenophobic narratives, and exclusionary policies intensify this marginalization, further limiting access to timely, high-quality maternal and newborn health services [15].

### 1.2. Public Charge Determination

In cases where public healthcare programs are technically available, many Latino families avoid them. Mixed-status households may be eligible for safety-net services but often fear that enrolling an eligible child or applying for nutrition or medical assistance could expose undocumented family members to immigration enforcement or create negative consequences that may jeopardize pathways to citizenship. The public charge rule, which requires non-citizens applying for legal status or residency to demonstrate that their use of public programs will not become dependent on government means of support, is often cited as creating a “chilling effect” in mixed status families, curbing their legal public benefit usage [15]. A public charge determination may bar those applying for visas or legal permanent resident status from entry to the United States or legal status. Under its original design, the 1999 Public Charge Rule deemed noncitizens a “public charge” if they received cash benefits (e.g., Supplemental Security Income) or government-financed long term institutional care [16]. As noted by the Department of Justice, this design was intended to avoid uncertainty regarding how being the recipient of public benefits affects immigration proceedings, thereby avoiding confusion that could deter individuals from accessing available benefits and negatively impact public health [17,18].

In 2019, the Department of Homeland Security (DHS) under the Trump administration strayed from this original design, expanding the public charge determination to include both cash benefits and public benefits such as non-emergency Medicaid, SNAP, and housing subsidies. Prior to the implementation of the 2019 rule, a draft of the executive order was leaked in January 2017, signaling the planned expansion of the public charge definition. Subsequent to this leak, studies documented significant participation decreases in SNAP (−7.3 percentage points) and National School Lunch Program (−12.6 percentage points) [19]. Consistent with these early responses, after implementation, prenatal care visits in the first trimester were reduced by 12% in immigrant mothers who were uninsured when compared to privately insured immigrants [20].

Public charge determination was later reversed by the Biden administration in 2020, and in 2022, DHS issued a rule that returned to a narrower, pre-Trump interpretation. In 2025, the Trump administration proposes to reverse this again. The newly proposed public charge rule would eliminate the 2022 limitations and remove any constraints on which public benefits may be considered, and gives immigration officers discretion to weigh past, current, or household benefit use in determinations [21]. This determination is critical because the finding of a public charge may outweigh otherwise satisfied criteria (e.g., marriage to a US citizen or Legal Permanent Resident; hardship to a qualifying relative; 10 years of good moral character, etc.) that an applicant otherwise met. The shifting policy and current proposal contribute to the confusion and deterrence that the original design sought to prevent.

These practices contradict international human rights commitments, which assert that access to essential healthcare should not depend on nationality, documentation status, or circumstances of migration [22]. This is especially true for pregnant women, where the benefits of prenatal care extend well beyond improvements in perinatal outcomes alone. Prenatal care serves as a key point of entry into the healthcare system, introducing and increasing the likelihood of continued lifetime preventive care and facilitating access to social and support services for mothers and children [23]. This integrative function of prenatal care is especially consequential for immigrant Latino families, for whom early health advantages at birth often deteriorate during early childhood [23]. This initial introduction may also connect individuals to pathways to postpartum care, childhood immunizations, and nutrition programs. Extensive evidence shows that the first 1000 days—from conception through a child’s second birthday—constitute a pivotal window in which maternal health, reproductive autonomy, exposure to stress, and the quality of medical care profoundly influence developmental, cognitive, and lifelong health outcomes [24].

## 2. Policy Mechanisms That Shape Access to MCH Healthcare and Public Health Services Among Latinos in the U.S.

Public health literature highlights racial and ethnic disparities in maternal and infant health, documenting widening gaps for Latina populations in infant mortality (within the first year), neonatal mortality (within the first 28 days), and adolescent mortality [25]. Recognized as a landmark year for Latinx migration as well as healthcare reform, 1965 marks the birth of the Immigration and Nationality Act (Hart-Celler Act) and the Medicare and Medicaid Act. The Immigration and Nationality Act imposed ceilings on immigration from Western Hemisphere countries for the first time and created the notion of an undocumented individual. However, it also created family reunification pathways to entry that bolstered Latinx families to migrate in swaths and settle in the U.S. for longer stays than in previous decades [26]. Simultaneously, the United States established Medicare and Medicaid, beginning a long journey towards an equitable federal healthcare plan for underserved populations in America. At its inception, Medicaid was available for legally present immigrants as it was for U.S.-born citizens if they met financial and demographic qualifications [27]. Medicaid access radically improved infant survival for low-income, non-White children in America, reduced maternal mortality significantly, and increased the likelihood of poor, non-White mothers having hospital births [28]. However, this precedent of improved access would change only a few decades later, complicating the immigrant relationship with healthcare access.

In 1972, the Women, Infants and Children (WIC) program was introduced, marking another pivotal moment in public health services in the U.S. WIC, a nutrition program focused on providing nutritious foods and supplementary dietary needs (like formula), began as a pilot program and was codified into law in 1975 with the goal of reducing maternal and child malnutrition in impoverished groups. Over the decades, WIC has expanded to include postpartum services, nutrition education, and breastfeeding resources and support, and has been proven to reduce poor infant and maternal health outcomes [29,30]. Notably, WIC does not require a mother to disclose immigration status and is therefore one of the few public benefits that all persons in America are eligible to receive, regardless of citizenship. Further, WIC is exempt from public charge determination, meaning it is currently protected from impacting the path to citizenship [31]. These protections mean immigrant mothers and children reap consistent benefits from WIC, including improved birthweight outcomes in babies born to immigrant mothers [31].

Immigrants deemed undocumented went without covered healthcare until 1986, when the Emergency Medical Treatment and Active Labor Act (EMTALA) introduced emergency Medicaid provisions, which required hospitals to provide emergency care to all individuals, regardless of legal status or coverage [32]. Further, EMTALA solidified healthcare coverage for pregnant women by giving states the option to cover all pregnant women with income below the federal poverty line [32,33]. With these expanded provisions, Medicaid-funded deliveries increased 17% from 1985 to 1991. However, EMTALA also sent a shock to the healthcare system by flooding emergency departments with people seeking routine care. Many hospitals experienced overcrowding and extreme financial strain, as coverage was not centrally organized to accommodate emergency visits for all people [34]. To this day, EMTALA remains a controversial piece of policy that is still the sole coverage for undocumented immigrants.

In 1996, the Personal Responsibility and Work Opportunity Reconciliation Act (PRWORA) and the Illegal Immigration Reform and Immigrant Responsibility Act (IIRIRA) created restrictive federal eligibility baselines. Most lawfully present immigrants became subject to a five-year waiting period for Medicaid, the Child Health Insurance Program (CHIP), and other major federal benefit programs. With the exception of emergency Medicaid, undocumented immigrants continued to be excluded from accessing these benefits. Administrative complexity and the implications of immigration surveillance further suppressed benefit enrollment [35]. After the 1996 policy reforms, immigrant enrollment in Medicaid dropped by 3% compared to the U.S. citizen’s 1.6% reduction [26]. Expanded immigration detention programs began to fracture families, separating mothers from children and causing severe psychological distress to undocumented and legal immigrants alike [36].

States attempted to mitigate the 1996 policy restrictions through state-only health insurance programs that provided prenatal (and in some places 12-month postpartum) coverage regardless of immigration status. The Children’s Health Insurance Plan (CHIP), established in 1997, supported these goals by allocating federal dollars towards low-income child healthcare. Specifically, CHIP’s Fetal and Children Health Expansion Program (FCEP) was established to provide pregnant mothers and unborn children coverage from conception, including legal permanent residents who would otherwise face the new five-year waiting period [35]. From 1997 to 2018, child uninsurance rates dropped from 14.2% to 5.5%, and several states have leveraged CHIP to serve populations that would not be covered by Medicaid, like immigrant mothers [37]. In 2009, the Children’s Health Insurance Program Reauthorization Act (CHIPRA) removed the five-year waiting period for certain lawfully residing immigrant children and pregnant women at the federal level, temporarily decreasing uninsured rates and increasing public insurance enrollment. However, these improvements diminished over time and were no longer statistically significant after three years [38].

From 2012 to 2015, high-profile detention of pregnant Latina migrants, anti-immigrant rhetoric, and expansion of enforcement programs, such as the Secure Communities Act, produced profound stressors and barriers to care. Secure Communities, a program designed to enable the federal government to check the immigration status of every single person arrested for a crime by local police, resulted in over 2.3 million arrests or convictions and 440,000 deportations, disproportionately affecting mothers and young children. One study found that the presence of the Secure Communities policy increased the incidence of very low birthweight by 21% in foreign-born Hispanic mothers, likely due to induced stress and undernutrition during pregnancy [25].

In 2018, the Zero Tolerance Policy was introduced, calling for the persecution of all people who crossed the border without inspection. The policy mandated criminal prosecution for all adults entering the United States without authorization, irrespective of prior criminal history, first-time entry status, the presence of accompanying children, or the existence of a valid asylum claim [39]. Notably, parents migrating with children were explicitly targeted, and asylum officers were directed to weigh unauthorized entry as a negative factor in asylum determinations. After apprehension at the border, families were processed in federal facilities where officials determined whether to pursue expedited removal, criminally prosecute the parent, or release the family to continue immigration proceedings in the United States outside of detention. In some cases, parents were misled or coerced into signing documents relinquishing their asylum rights [39]. Separation from parents is among the most traumatic experiences a child can endure and has been consistently linked to increased risk of anxiety, depression, post-traumatic stress, sleep disturbances, behavioral problems, and poorer educational outcomes. Evidence from studies of migration-related separation and parental deportation indicates that the sudden, prolonged, and uncertain nature of these separations—particularly when a primary caregiver is removed—disrupts healthy social and emotional development and is associated with more severe and lasting mental health consequences for affected children.

In the 2020s, noncitizen immigrants remain more likely to be uninsured due to job-based coverage gaps and eligibility restrictions. Lawfully present groups still face the five-year waiting period for Medicaid/CHIP (with state and CHIP prenatal options as partial workarounds), and undocumented immigrants are excluded from federally funded coverage except Emergency Medicaid [13]. A 2025 CMS policy letter clarified that Emergency Medicaid does not encompass managed-care capitation payments, narrowing reimbursement pathways for hospitals serving “aliens ineligible for full Medicaid benefits” [40]. Historically, Section 1011 offered limited hospital reimbursement for emergency care to undocumented patients [41]. Recent federal changes further restrict The Patient Protection and Affordable Care Act (ACA) subsidies and other federal benefits for some lawfully present groups, eliminating temporary coverage flexibilities that once allowed up to 90 days of care during documentation processing [42].

Furthermore, if the proposed public charge rule, previously mentioned, comes to fruition, immigration officers would be permitted to consider any public benefit used by an individual or members of their household, removing prior regulatory language that excluded benefits received by family members and offering no meaningful guardrails for how those benefits should be weighed [43]. This expanded discretion is particularly consequential for Latino families, who are more likely to live in mixed-status households and rely on safety-net programs during periods of economic instability, pregnancy, or early childhood. Although DHS emphasizes the importance of accuracy and consistency in adjudications, the absence of concrete guidance virtually guarantees unpredictable outcomes; for example, one officer might deem a parent inadmissible based on temporary household use of Medicaid, while another might not. DHS has also indicated that future modifications may be shaped through internal memoranda and manuals, further increasing uncertainty for families attempting to make decisions about health care, nutrition, and housing [21]. By reintroducing ambiguity and expanding the scope of consideration beyond the already harmful 2019 rule, the 2025 proposal is likely to intensify the chilling effect, deterring Latino families from accessing essential services and undermining household stability, maternal health, and early childhood development—even among those who are fully eligible for assistance [15,44,45].

Taken together, these historical and contemporary policy shifts reveal a consistent pattern: access to maternal and child health services for Latino families has been shaped not only by clinical need, but by immigration status, enforcement priorities, and changing eligibility rules. Periods of expanded coverage and inclusion have been associated with measurable improvements in maternal and infant outcomes, while restrictive policies, enforcement-driven family separation, and benefit exclusions have generated fear, reduced participation in safety-net programs, and contributed to preventable disparities. The cumulative effect of this fragmented and unstable policy environment is a system in which access to essential services for women and children is inconsistent, uncertain, and often contingent on legal status rather than health need. These findings point to the urgent need for policy solutions that stabilize access to care, reduce deterrent effects, and center maternal and child health in immigration-related decision-making.

## 3. Policy Recommendations to Protect Latina Maternal–Child Health

Building on this historical and policy analysis, we outline two targeted recommendations designed to reduce harm, protect foundational health resources, and align immigration policy with longstanding public health and human rights principles. For Latino families, exclusions and fear embedded in current policy frameworks disrupt access to essential services, undermine trust in health and social systems, and place women and children at heightened risk during critical periods of development. Addressing these challenges requires policy solutions that move beyond narrow immigration considerations to explicitly center on maternal and child health. We provide two policy recommendations that outline concrete, actionable strategies to reduce harm, protect foundational health resources, and align immigration policy with long-standing public health and human rights principles.

### 3.1. Policy Recommendation 1: Restore the Public Charge Determination to Its Original, Limited Design

We recommend that the public charge determination be permanently returned to its original framework, in which only cash assistance and government-financed long-term institutional care are considered, and participation in public safety-net programs is explicitly excluded. Expansions of the public charge rule to include noncash benefits—such as Medicaid, SNAP, or housing assistance—have repeatedly generated chilling effects, discouraging immigrant families from accessing essential maternal and child health services even when legally eligible. These deterrent effects are particularly harmful during pregnancy and early childhood, when timely access to care and nutrition is critical [44,45].

Public charge policy should clearly insulate the use of health, nutrition, and family-support programs from adverse immigration consequences. As implemented under previous federal administrations, statutory language and agency guidance should specify that participation in Medicaid (including pregnancy-related coverage), WIC, SNAP, and other foundational safety-net programs cannot be weighed negatively in admissibility, adjustment-of-status, or discretionary determinations [43]. Restoring and stabilizing the original design would reduce confusion, rebuild trust in public institutions, and align immigration policy with longstanding public health principles that prioritize the health of women and children [43].

### 3.2. Policy Recommendation 2: Waive the Five-Year Medicaid Waiting Period for All Pregnant Immigrants, Regardless of Documentation Status

Given the well-documented importance of prenatal and postpartum care for maternal and infant health, we recommend that all 50 states waive the five-year Medicaid waiting period for pregnant immigrants, and extend pregnancy-related Medicaid coverage regardless of immigration status [46,47]. At present, such waivers are available in only 31 states and apply primarily to certain categories of lawful noncitizens, leaving substantial gaps in coverage nationwide. This recommendation is in alignment with previous legislative proposals introduced by the 118th Congress, specifically the LIFT The BAR Acti (H.R. 4170). The bill sought to reappeal limits on means-tested federal benefits for individuals who were identified as lawful, noncitizens of the U.S [48]. Current eligibility restrictions and state-by-state variability create fragmented coverage that excludes many pregnant people from timely prenatal care and limits postpartum coverage during a period of heightened risk for maternal morbidity and mortality [49,50].

Universal pregnancy-related Medicaid coverage would promote continuity of care across the reproductive life course, reduce preventable complications, and help address persistent racial and ethnic disparities in maternal outcomes [50]. Furthermore, Medicaid expansions for pregnant women not only improve birth outcomes for the first generation but have also yielded persistent intergenerational health benefits, with the children of women exposed to expanded coverage in utero experiencing better health outcomes [51]. Extending eligibility during pregnancy and the postpartum period is not only a cost-effective public health intervention but also a critical step toward ensuring that immigration status does not determine access to essential, life-saving care.

## 4. Conclusions

When access to health care and public health services is conditioned on immigration status, maternal and child health outcomes in Latino communities are disproportionately harmed. Although this is not a systematic review, this analytic policy commentary highlights key federal and state policies that directly shape maternal and child health outcomes among Latino families. Fragmented eligibility rules, exclusionary policies, and fear of immigration-related consequences limit access to timely prenatal, postpartum, and pediatric care during critical periods of development, undermining the health of both women and children. These barriers contribute to preventable morbidity, widen existing health disparities, and perpetuate intergenerational inequities that extend beyond individual families to affect communities and health systems more broadly. Addressing these challenges requires policy reforms that decouple access to essential maternal and child health services from immigration enforcement, prioritize equity and family stability, and align immigration policy with established public health and human rights principles.

## Figures and Tables

**Table 1:** Public Benefit Eligibility by Immigration Status.

Immigration Status	Medicaid + Chip	SNAP	TANF	SSI	WIC	Emergency Medicaid
**US Born Citizen (USC):** all persons born in the United States.	X	X	X	X	X	X
**Naturalized Citizen:** foreign-born nationals who complete the process to voluntarily become a US citizen, usually after a 3–5 year waiting period as an LPR.	X	X	X	X	X	X
**Lawful/Legal Permanent Resident (LPR):** non-citizens who have permission to live and work in the United States indefinitely; green card holders.	X ^1^	X ^1^	X ^1^	X	X	X
**Conditional Permanent Resident:** non-citizens who have been lawfully admitted for permanent residence, subject to specified conditions and responsibilities.	X ^2^		X ^1^	X	X	X
**Family Sponsored Visa:** non-citizens who seek to live permanently in the US and are sponsored by a US citizen or LPR spouse, child, parent, or sibling.	X ^1^		X ^3^	X ^3^	X	X
**Battered Immigrant Status/Violence Against Women Act (VAWA) Sponsors:** non-citizen spouse, fiancée, former spouse, or child of an abusive USC or LPR partner or parent.	X ^4^		X ^1^	X	X	X
**Special Immigrant Juvenile:** non-citizens under the age of 21, whom a family court deemed remaining in the US to be in their best interest, and cannot return to their home country due to abuse, neglect or abandonment by a parent.			X ^1^		X	X
**Refugee/Asylee:** a non-citizen who is unable or unwilling to return to their home country of nationality because of past persecution, or a well-founded fear of future persecution, based on their race, religion, nationality, political opinion, or membership in a particular social group.	X		X	X	X	X
**Non-Immigrant Temp Visas:** non-citizens who are admitted to the US for a limited period and a specific purpose.					X	X
**T Nonimmigrant Visa:** immigration benefit for victims of severe forms of human trafficking.	X ^5^		X	X	X	X
**Deferred Enforced Departure (DED):** authorization from the president which halts removal of a noncitizen, generally for a designated period.					X	X
**Deferred Action for Childhood Arrivals (DACA):** non-citizens who were brought to the US as children and granted temporary protection from deportation.					X	X
**Temporary Protected Status (TPS):** non-citizens from specific foreign countries, which are experiencing challenges that make it difficult or unsafe for the noncitizen to be returned.			X ^6^		X	X
**Withholding of Deportation:** if barred from applying for asylum, but still able to prove a well-founded fear of persecution based on a protected ground, the court may issue an order of removal and prohibit the government from executing the order; the noncitizen may still be removed to a third country.	X		X	X	X	X
**Cuban and Haitian Entrants:** Cuban or Haitian nationals who are granted entry into the United States but do not have permanent status.	X	X	X	X	X	X
**Undocumented Immigrant:** non-citizens who enter the country without permission or, have legally entered on a valid nonimmigrant visa but whose visa has expired.					X	X

^1^ Subject to five-year waiting period; ^2^ For applicants before 1980; ^3^ Dependent on sponsor; ^4^ Subject to five-year waiting period or proof of immediate eligibility; ^5^ Includes pending application; ^6^ Dependent on state.

## Data Availability

No new data were created or analyzed in this study.

## Abbreviations

The following abbreviations are used in this manuscript:

MCH	Maternal and child health
CHIP	Child Health Insurance Program
TANF	Temporary Assistance for Needy Families
DACA	Deferred Action Childhood Arrival
TPS	Temporary Protected Status
USC	US Born Citizen
LPR	Lawful/Legal Permanent Resident
VAWA	Violence Against Women Act
DED	Deferred Enforced Departure
DHS	Department of Homeland Security
SNAP	Supplemental Nutrition Assistance Program
WIC	Women, Infants, and Children
EMTALA	Emergency Medical Treatment and Active Labor Act
PRWORA	Personal Responsibility and Work Opportunity Reconciliation Act
FCEP	Fetal and Children Health Expansion Program
CHIPRA	Children’s Health Insurance Program Reauthorization Act
